# Neurological outcomes in patients transported to hospital without a prehospital return of spontaneous circulation after cardiac arrest

**DOI:** 10.1186/cc13121

**Published:** 2013-11-20

**Authors:** Yoshikazu Goto, Tetsuo Maeda, Yumiko Nakatsu-Goto

**Affiliations:** 1 Section of Emergency Medicine, Kanazawa University Hospital, 13-1 Takaramachi, Kanazawa 920-8641, Japan; 2 Department of Cardiology, Yawata Medical Center, 12-7 I Yawata, Komatsu 923-8551, Japan

## Abstract

**Introduction:**

As emergency medical services (EMS) personnel in Japan are not allowed to perform termination of resuscitation in the field, most patients experiencing an out-of-hospital cardiac arrest (OHCA) are transported to hospitals without a prehospital return of spontaneous circulation (ROSC). As the crucial prehospital factors for outcomes are not clear in patients who had an OHCA without a prehospital ROSC, we aimed to determine the prehospital factors associated with 1-month favorable neurological outcomes (Cerebral Performance Category scale 1 or 2 (CPC 1–2)).

**Methods:**

We analyzed the data of 398,121 adult OHCA patients without a prehospital ROSC from a prospectively recorded nationwide Utstein-style Japanese database from 2007 to 2010. The primary endpoint was 1-month CPC 1–2.

**Results:**

The rate of 1-month CPC 1–2 was 0.49%. Multivariate logistic regression analysis indicated that the independent variables associated with CPC 1–2 were the following nine prehospital factors: (1) initial non-asystole rhythm (ventricular fibrillation (VF): adjusted odds ratio (aOR), 9.37; 95% confidence interval (CI), 7.71 to 11.4; pulseless ventricular tachycardia (VT): aOR, 8.50; 95% CI, 5.36 to 12.9; pulseless electrical activity (PEA): aOR, 2.75; 95% CI, 2.40 to 3.15), (2) age <65 years (aOR, 3.90; 95% CI, 3.28 to 4.67), (3) arrest witnessed by EMS personnel (aOR, 2.82; 95% CI, 2.48 to 3.19), (4) call-to-hospital arrival time <24 minutes (aOR, 2.58; 95% CI, 2.22 to 3.01), (5) arrest witnessed by any layperson, (6) physician-staffed ambulance, (7) call-to-response time <5 minutes, (8) prehospital shock delivery, and (9) presumed cardiac cause. When four crucial key factors (with an aOR >2.0 in the regression model: initial non-asystole rhythm, age <65 years, EMS-witnessed arrest, and call-to-hospital arrival time <24 minutes) were present, the rates of 1-month CPC 1–2 and 1-month survival were 16.1% and 23.2% in initial VF, 8.3% and 16.7% in pulseless VT, and 3.8% and 9.4% in PEA, respectively.

**Conclusions:**

In OHCA patients transported to hospitals without a prehospital ROSC, nine prehospital factors were significantly associated with 1-month CPC 1–2. Of those, four are crucial key factors: initial non-asystole rhythm, age <65 years, EMS-witnessed arrest, and call-to-hospital arrival time <24 minutes.

## Introduction

Out-of-hospital cardiac arrest (OHCA) is an increasing public health concern in industrial countries [1-3]. Despite decades of research, the survival rates after an OHCA have remained virtually unchanged in the last three decades [3]. The outcomes of patients with OHCA are associated with a multitude of variables, including age, comorbidities, initial recorded cardiac rhythm, and other circumstances related to cardiac arrest, such as the time to return of spontaneous circulation (ROSC) [4].

Two termination-of-resuscitation rules [5,6] for emergency medical services (EMS) personnel in prehospital settings have been introduced worldwide to predict survival from OHCA. These rules include five prehospital predictors: arrest witnessed by a bystander, arrest witnessed by EMS personnel, provision of cardiopulmonary resuscitation (CPR) by a bystander, shockable cardiac rhythm, and ROSC in the field. By far the most powerful criterion associated with survival from OHCA is ROSC in the field, irrespective of the sophistication of the subsequent in-hospital care [3]. However, the crucial prehospital factors for long-term survival with meaningful neurological outcomes in OHCA patients transported to hospitals without prehospital ROSC are not clear.

This study aimed to determine the prehospital factors that influence the 1-month favorable neurological outcomes in patients transported to hospitals without a prehospital ROSC after an OHCA.

## Materials and methods

### Study design and data source

The present investigation was a nationwide population-based observational study of all adult patients (age ≥18 years) for whom resuscitation had been attempted after an OHCA in Japan between January 1 2007 and December 31 2010. Cardiac arrest was defined as the cessation of cardiac mechanical activities, as confirmed by the absence of signs of circulation [1]. The cause of arrest was presumed to be cardiac unless evidence suggested external causes (trauma, hanging, drowning, drug overdose, or asphyxia), respiratory diseases, cerebrovascular diseases, malignant tumors, or any other non-cardiac cause. The attribution of cause as non-cardiac or cardiac was made by the physicians in charge in collaboration with the EMS personnel. This study was approved by the Ethical Committee of Kanazawa University. According to the informed consent guidelines in Japan [7], it is unnecessary to have informed consent from each patient to use secondary data such as on this anonymous database. Therefore, the requirement for written informed consent was waived.

### EMS system in Japan

Japan has approximately 127 million residents in an area of 378,000 km^2^, approximately two-thirds of which is uninhabited mountainous terrain [1,8,9]. Details about the Japanese EMS system have been described previously [1,8,9]. Briefly, municipal governments provide EMS through approximately 800 fire stations with dispatch centers. The Fire and Disaster Management Agency (FDMA) of Japan supervises the nationwide EMS system [1,8,9], whereas each local EMS system is operated by the local fire station. Generally, an ambulance crew includes three EMS staff, including at least one emergency life-saving technician (ELST) [1,9]. ELSTs are allowed to use several resuscitation methods, including use of semi-automated external defibrillators, insertion of a supraglottic airway device (laryngeal mask airway, laryngeal tube, and esophageal-tracheal twin-lumen airway device), insertion of a peripheral intravenous line, and administration of Ringer lactate solution [1,9]. Since July 2004, only specially trained ELSTs are permitted to insert a tracheal tube, and since April 2006 they have been permitted to administer intravenous epinephrine in the field under the instruction of an online physician [1,9]. All EMS providers perform CPR according to the Japanese CPR guidelines [10], based on the 2005 American Heart Association guidelines [11], since October, 2006. As EMS personnel in Japan are legally prohibited from terminating resuscitation in the field, most OHCA patients receive CPR from EMS providers and are transported to hospitals, except in cases where fatality is certain [1]. Epinephrine use is implemented according to the FDMA resuscitation guidelines for ELSTs [10,12]. The guidelines allow ELSTs to attempt intravenous access only twice, and each attempt must take no longer than 90 s. The allowed dosage of epinephrine is 1 mg per attempt, and repeated doses may be administered under a physician’s instruction.

### Data collection and quality control

The FDMA launched a prospective population-based observational study including all OHCA patients who received EMS in Japan since January 2005 [1,9]. EMS personnel at each center recorded the data for OHCA patients with the cooperation of the physician in charge, using an Utstein-style template [13]. The data were transferred to their fire stations and were then integrated into the registry system on the FDMA database server. The data were checked for consistency by the computer system and were confirmed by the FDMA. If the data form was incomplete, the FDMA returned it to the respective fire station, and the form was completed [1]. All data were stored in the nationwide database developed by the FDMA for public use. The FDMA provided permission to analyze this database and provided all the anonymous data to our research group. The main items included in the dataset were as follows: sex, age, causes of arrest (presumed cardiac origin or not), bystander witness status, bystander CPR with or without automated external defibrillator use, initial identified cardiac rhythm, bystander category (that is, the presence or absence of a bystander, or whether the bystander was a layperson or EMS personnel), whether epinephrine was administered, whether advanced airway management techniques (including endotracheal tube, laryngeal mask airway, and esophageal-tracheal tube) were used, whether ROSC was attained before arrival at the hospital, time of the emergency call, time of vehicle arrival at the scene, time of ROSC, time of vehicle arrival at the hospital, time of epinephrine administration, 1-month survival, and neurological outcome at 1 month after cardiac arrest. The neurological outcome was defined using the cerebral performance category (CPC) scale as follows: category 1, good cerebral performance; category 2, moderate cerebral disability; category 3, severe cerebral disability; category 4, coma or vegetative state; and category 5, death [13]. The CPC categorization was determined by the physician in charge. The call-to-response time was calculated as the time from the emergency call to the time of vehicle arrival at the scene. The call-to-hospital arrival time was calculated as the time from the emergency call to the time of vehicle arrival at the hospital.

### End points

The primary study end point was 1-month favorable neurological outcome (defined as a CPC of 1 or 2) [13]. The secondary end point was survival at 1 month after the OHCA.

### Statistical analysis

Kolmogorov-Smirnov-Lilliefors tests were performed to evaluate the distributions of continuous variables, and we found that all continuous variables were not normally distributed (all *P*-values <0.01). Therefore, the Wilcoxon and Kruskal-Wallis tests for continuous variables and the chi-square test for categorical variables were performed to compare the characteristics or outcomes between the cohorts. Multivariate logistic regression analysis including 14 variables was performed to assess the factors contributing to 1-month survival and 1-month CPC 1 to 2 for all eligible patients. The 14 selected variables included year, age, sex, arrest witnessed by any layperson, arrest witnessed by EMS personnel, bystander CPR, presumed cause of arrest, initial cardiac rhythm, prehospital shock delivery, advanced airway management, physician-staffed ambulance, call-to-response time, call-to-hospital arrival time, and prehospital epinephrine administration for the model as an independent variable. These models yielded concordance statistics of 0.78 for 1-month survival and 0.84 for 1-month CPC 1 to 2, which indicated good discrimination.

In the multivariate logistic regression analysis of outcomes, we classified the following three continuous variables into three categories according to the IQR of each variable: age (<65 years, 65 to 85 years, >85 years), call-to-response time (<5 minutes, 5 to 9 minutes, >9 minutes), and call-to-hospital arrival time (<24 minutes, 24 to 37 minutes, >37 minutes).

Continuous variables are expressed as means and standard deviations. Categorical variables are expressed as percentages. As an estimate of effect size and variability, we report odds ratios (ORs) with 95% (CIs. All statistical analyses were performed using the JMP statistical package version 10 (SAS Institute Inc., Cary, NC, USA). All tests were two-tailed, and a value of *P* <0.05 was considered statistically significant.

## Results

**Figure 1:**
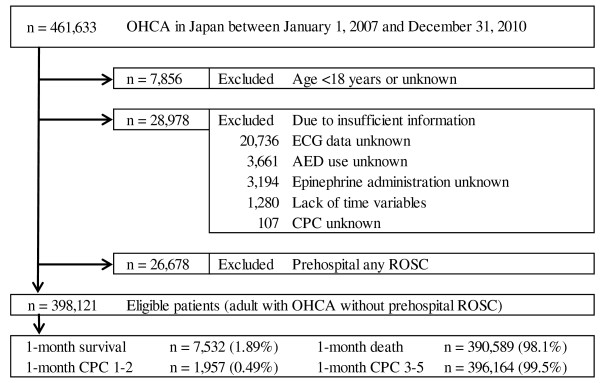
**Study profile with the selection of participants.** AED, automated external defibrillator; CPC, cerebral performance category; ECG, electrocardiogram; OHCA, out-of-hospital cardiac arrest; ROSC, return of spontaneous circulation.

During the 4-year study period, 461,633 patients were documented in the database. We excluded patients with any prehospital ROSC, no matter how transient, and finally considered 398,121 (86.2%) patients eligible for enrollment into this study. Figure 1 shows a flow diagram depicting the inclusion and exclusion criteria for subjects in the present study. The overall 1-month survival and favorable neurological outcome (CPC 1 to 2) rates were 1.89% (n = 7,532) and 0.49% (n = 1,957), respectively.

Table 1 shows the baseline characteristics of study patients according to 1-month survival after OHCA. No significant difference was found in year and the use of advanced airway management. Age, call-to-response time, and call-to-hospital arrival time in the survival cohort were significantly lower than those in the death cohort (*P* <0.0001). The values of the other nine variables in the survival cohort were significantly higher than those in the 1-month death cohort (all *P* <0.0001, except bystander CPR with *P* = 0.027).

**Table 1:** Baseline characteristics of the study patients according to 1-month survival

**Characteristic**	**Survival (n = 7,532)**	**Death (n = 390,589)**	***P-*** **value**
Year			
2007	1,734 (23.0)	89,078 (22.8)	0.7152
2008	1,899 (25.2)	97,715 (25.0)	
2009	1,861 (24.7)	98,802 (25.3)	
2010	2,038 (27.1)	104,994 (26.9)	
Age, years (median 77, IQR 65 to 85)	69.0 ± 16.2	73.8 ± 16.2	<0.0001
< 65	2,632 (34.9)	91,697 (23.5)	<0.0001
65 to 85	3,875 (51.5)	204,762 (52.4)	
> 85	1,025 (13.6)	94,130 (24.1)	
Male	4,743 (63.0)	224,814 (57.6)	<0.0001
Arrest witnessed by any layperson	5,112 (67.9)	140,948 (36.1)	<0.0001
Arrest witnessed by EMS personnel	1,039 (13.8)	17,562 (4.5)	<0.0001
Bystander CPR	3,429 (45.5)	172,815 (44.2)	0.0266
Presumed cardiac cause	4,444 (59.0)	218,477 (55.9)	<0.0001
Initial cardiac rhythm			
Ventricular fibrillation	2,144 (28.5)	21,352 (5.5)	<0.0001
Pulseless ventricular tachycardia	54 (0.7)	673 (0.2)	
Pulseless electrical activity	2,696 (35.8)	81,312 (20.8)	
Asystole	2,638 (35.0)	287,252 (73.5)	
Prehospital actual shock delivery	2,311 (30.7)	33,809 (8.7)	<0.0001
Use of advanced airway management	2,414 (32.1)	125,583 (32.2)	0.889
Epinephrine administration	698 (9.3)	29,519 (7.6)	<0.0001
Physician-staffed ambulance	282 (3.7)	8,132 (2.1)	<0.0001
Call-to-response time, minutes (median 7, IQR 5 to 9)	6.6 ± 3.1	7.5 ± 3.8	<0.0001
< 5	1,608 (21.4)	63,019 (16.1)	<0.0001
5 to 9	5,014 (66.6)	248,769 (63.7)	
> 9	910 (12.1)	78,801 (20.2)	
Call-to-hospital arrival time, minutes (median 30, IQR 24 to 37)	27.8 ± 11.1	31.9 ± 11.7	<0.0001
< 24	2,808 (37.3)	86,614 (22.2)	<0.0001
24 to 37	3,817 (50.7)	216,927 (55.5)	
> 37	907 (12.0)	87,048 (22.3)	

Values are reported either as n (%) or mean ± standard deviation. CPC, cerebral performance category; CPR, cardiopulmonary resuscitation; EMS, emergency medical service.

Table 2 shows the baseline characteristics of the study patients according to 1-month neurological outcomes after OHCA. In the CPC 1 to 2 cohort, patients were approximately 10 years younger and a higher proportion was male, as well as higher rates of bystander-witnessed arrest, EMS-witnessed arrest, bystander CPR, presumed cardiac cause, ventricular fibrillation (VF), prehospital actual shock delivery, and physician-staffed ambulance than those in the CPC 3 to 5 cohort (all *P* <0.0001, except bystander CPR with *P* = 0.009). The number of times advanced airway management was used in the CPC 1 to 2 cohort was significantly less than that in the CPC 3 to 5 cohort (*P* <0.0001). Call-to-response and call-to-hospital arrival times in the CPC 1 to 2 cohort were significantly shorter that those in the CPC 3 to 5 cohort by approximately 1 minute and 4 minutes, respectively. There were no significant differences between the two cohorts in the year or prehospital epinephrine administration.

**Table 2:** Baseline characteristics of the study patients according to 1-month neurological outcomes

**Characteristic**	**CPC 1 to 2 (n = 1,957)**	**CPC 3 to 5 (n = 396,164)**	***P-*** **value**
Year			
2007	428 (21.9)	90,384 (22.8)	0.391
2008	508 (26.0)	99,106 (25.0)	
2009	475 (24.3)	100,188 (25.3)	
2010	546 (27.9)	106,486 (26.9)	
Age, years (median 77, IQR 65 to 85)	64.4 ± 16.3	73.7 ± 16.2	<0.0001
< 65	938 (47.9)	93,391 (23.6)	<0.0001
65 to 85	861 (44.0)	207,776 (52.4)	
> 85	158 (8.1)	94,997 (24.0)	
Male	1,374 (70.2)	228,183 (57.6)	<0.0001
Arrest witnessed by any layperson	1,430 (73.1)	144,630 (36.5)	<0.0001
Arrest witnessed by EMS personnel	398 (20.3)	18,203 (4.6)	<0.0001
Bystander CPR	924 (47.2)	175,320 (44.3)	0.009
Presumed cardiac cause	1,431 (73.1)	221,490 (55.9)	<0.0001
Initial cardiac rhythm			
Ventricular fibrillation	922 (47.1)	22,574 (5.7)	<0.0001
Pulseless ventricular tachycardia	24 (1.2)	703 (0.2)	
Pulseless electrical activity	573 (29.3)	83,435 (21.1)	
Asystole	438 (22.4)	289,452 (73.1)	
Prehospital actual shock delivery	938 (47.9)	35,182 (8.9)	<0.0001
Use of advanced airway management	502 (25.7)	127,497 (32.2)	<0.0001
Epinephrine administration	168 (8.6)	30,049 (7.6)	0.10
Physician-staffed ambulance	96 (4.9)	8,318 (2.1)	<0.0001
Call-to-response time, minutes (median 7, IQR 5 to 9)	6.4 ± 3.2	7.5 ± 3.8	<0.0001
< 5	468 (23.9)	64,159 (16.2)	<0.0001
5 to 9	1,290 (65.9)	252,493 (63.8)	
> 9	199 (10.2)	79,512 (20.1)	
Call-to-hospital arrival time, minutes (median 30, IQR 24 to 37)	28.1 ± 12.8	31.8 ± 11.6	<0.0001
< 24	774 (39.5)	88,648 (22.4)	<0.0001
24 to 37	917 (46.9)	219,827 (55.5)	
> 37	266 (13.6)	87,689 (22.1)	

Values are reported either as n (%) or mean ± standard deviation. CPC, cerebral performance category; CPR, cardiopulmonary resuscitation; EMS, emergency medical service.

Table 3 shows the results of multivariate logistic regression analysis including 14 variables to determine the factors associated with 1-month survival and 1-month CPC 1 to 2. Ten of the fourteen variables were associated with increased odds of survival. The highest adjusted OR was initial VF rhythm with an OR of 5.50 (95% CI 4.96, 6.10) compared with initial asystole rhythm. Other initial non-asystole rhythm types (pulseless ventricular tachycardia (VT): OR 4.58, 95% CI 3.40, 6.05; pulseless electrical activity (PEA): OR 2.38, 95% CI 2.24, 2.52) also had a strong association with survival followed by call-to-hospital arrival time <24 minutes, arrest witnessed by any layperson, and age <65 years. Of the fourteen variables, nine variables were associated with increased odds of CPC 1 to 2. The highest adjusted OR was initial VF rhythm with an OR of 9.37 (95% CI 7.71, 11.4) compared with initial asystole rhythm. Other initial non-asystole rhythm types (pulseless VT: OR 8.50, 95% CI 5.36, 12.9; PEA: OR 2.75, 95% CI 2.40, 3.15) also had a strong association with survival followed by age <65 years, arrest witnessed by EMS personnel, and call-to-hospital arrival time <24 minutes.

**Table 3:** Results of multivariate logistic regression analyses for variables associated with 1-month outcomes

	**Adjusted odds ratio (95% CI)**	
**Variables**	**One-month survival**	**One-month CPC 1 to 2**
Year		
2007	Reference	Reference
2008	1.04 (0.97, 1.11)	1.12 (0.98, 1.28)
2009	1.03 (0.97, 1.11)	1.07 (0.93, 1.22)
2010	1.15 (1.07, 1.24)	1.15 (1.00, 1.31)
Age, years		
< 65	2.13 (1.97, 2.30)	3.90 (3.28, 4.67)
65 to 85	1.59 (1.48, 1.71)	2.00 (1.69, 2.40)
> 85	Reference	Reference
Male	0.89 (0.85, 0.94)	0.95 (0.86, 1.06)
Arrest witnessed by any layperson	2.15 (2.04, 2.28)	1.90 (1.69, 2.13)
Arrest witnessed by EMS personnel	1.73 (1.60, 1.86)	2.82 (2.48, 3.19)
Bystander CPR	1.00 (0.96, 1.05)	1.04 (0.95, 1.14)
Presumed cardiac cause	0.87 (0.83, 0.91)	1.41 (1.26, 1.57)
Initial cardiac rhythm		
Ventricular fibrillation	5.50 (4.96, 6.10)	9.37 (7.71, 11.4)
Pulseless ventricular tachycardia	4.58 (3.40, 6.05)	8.50 (5.36, 12.9)
Pulseless electrical activity	2.38 (2.24, 2.52)	2.75 (2.40, 3.15)
Asystole	Reference	Reference
Prehospital actual shock delivery	1.40 (1.28, 1.54)	1.61 (1.35, 1.91)
Use of advanced airway management	1.14 (1.07, 1.20)	0.79 (0.71, 0.89)
Epinephrine administration	0.97 (0.89, 1.05)	0.79 (0.67, 0.94)
Physician-staffed ambulance	1.46 (1.28, 1.65)	1.68 (1.35, 2.07)
Call-to-response time, minutes		
< 5	1.29 (1.18, 1.41)	1.67 (1.40, 2.00)
5 to 9	1.24 (1.15, 1.34)	1.46 (1.25, 1.71)
> 9	Reference	Reference
Call-to-hospital arrival time, minutes		
< 24	3.19 (2.93, 3.46)	2.58 (2.21, 3.01)
24 to 37	1.74 (1.62, 1.88)	1.37 (1.19, 1.58)
> 37	Reference	Reference

CPC, cerebral performance category; CPR, cardiopulmonary resuscitation; EMS, emergency medical service.

**Figure 2:**
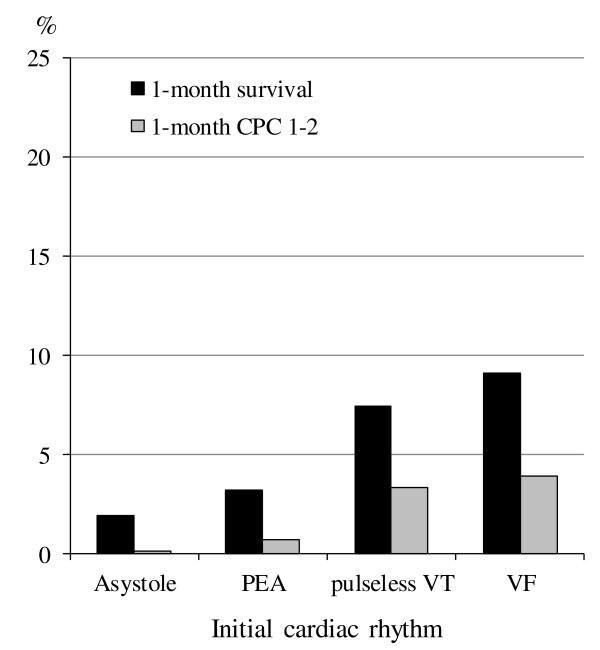
**Crude 1-month outcomes according to the initial rhythm.** CPC, cerebral performance category; PEA, pulseless electrical activity; VF: ventricular fibrillation; VT: ventricular tachycardia.

Figure 2 shows the rates of crude 1-month survival and 1-month CPC 1 to 2 according to the initial cardiac rhythm. The rates of 1-month CPC 1 to 2 and 1-month survival were 3.9% and 9.1% in initial VF, 3.3% and 7.4% in initial pulseless VT, 0.68% and 3.2% in initial PEA, and 0.15% and 1.89% in initial asystole, respectively.

**Figure 3:**
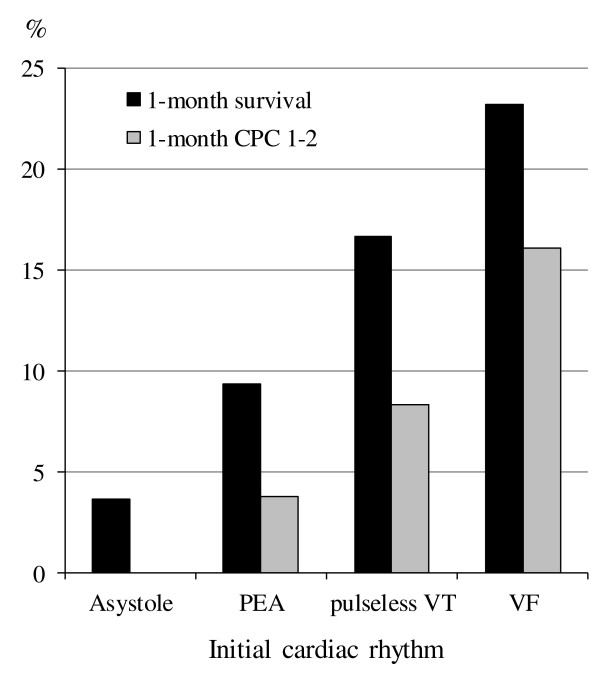
**One-month outcomes according to the initial rhythm in the presence of four crucial key factors.** CPC, cerebral performance category; PEA, pulseless electrical activity; VF: ventricular fibrillation; VT: ventricular tachycardia.

We selected a prehospital variable as a crucial key prehospital factor when the adjusted OR of each variable was >2.0 in the multivariate logistic regression model for 1-month CPC 1 to 2. There were four crucial key factors that met the criterion: initial non-asystole rhythm (VF, pulseless VT, and PEA), age <65 years, arrest witnessed by EMS personnel, and call-to-hospital arrival time <24 minutes. Figure 3 shows the rates of 1-month outcomes according to the initial rhythm when all four crucial key prehospital factors were present. The rates of 1-month CPC 1 to 2 and 1-month survival were 16.1% and 23.2% in initial VF, 8.3% and 16.7% in initial pulseless VT, 3.8% and 9.4% in initial PEA, and 0% and 3.64% in initial asystole, respectively.

## Discussion

In OHCA patients transported to hospitals without a prehospital ROSC, the present analysis of >398,000 adult patients with OHCA in Japan demonstrates that the most crucial prehospital variable for favorable neurological outcomes at 1 month after OHCA was initial non-asystole rhythm (VF, pulseless VT, and PEA) followed by age <65 years, arrest witnessed by EMS personnel, and call-to-hospital arrival time <24 minutes. When all these four crucial key predictors for favorable neurological outcomes were present, approximately one of every six patients with initial VF rhythm had 1-month favorable neurological outcomes after OHCA without prehospital ROSC.

As EMS personnel in Japan are not allowed to perform termination of resuscitation in the field [10], 93.7% (398,121 of 424,799) of patients experiencing OHCA are transported to hospital without a prehospital ROSC. This enabled us to investigate in the present study a large number of OHCA patients without a prehospital ROSC. A meta-analysis by Sasson *et al*. [3] indicated that ROSC in the field is the most powerful factor associated with survival from OHCA followed by EMS-witnessed arrest, initial VF/pulseless VT, bystander CPR, bystander-witnessed arrest, and initial non-asystole rhythm. In their paper, the pooled OR for survival to hospital discharge in patients who achieved ROSC in the field (versus those who did not) ranged from 20.96 (95% CI 7.43, 59.13) to 99.84 (95% CI 14.30, 696.89) according to the baseline survival rates, and the rate for survival to discharge after OHCA without prehospital ROSC ranged from 0.1% to 1.8%. Therefore, the absence of prehospital ROSC indicates that patients with OHCA will not likely survive or will not have favorable neurological outcomes. As our 1-month survival rate of OHCA patients without prehospital ROSC was 1.89%, our results were consistent with high baseline survival-rates in the study cohort of Sasson *et a*l. [3]. Even in such difficult conditions, the presence of the above-mentioned four key factors would increase the rates of survival and favorable neurological outcomes at 1 month after an OHCA. Especially, the rate of 1-month CPC 1 to 2 in initial VF patients who also had the other three key factors was approximately 4-fold higher than that in patients who did not have those factors (Figures 2 and 3).

The most important factor for an increased chance of favorable neurological outcomes was initial non-asystole rhythm, especially VF rhythm in OHCA patients without prehospital ROSC. The percentage of initial VF rhythm in our study subjects was 5.9% (n = 23,496). The crude ORs of initial VF for the association with 1-month survival and 1-month CPC 1 to 2 (versus non-VF) were 6.88 (95% CI 6.53, 7.25) and 14.74 (95% CI 13.48, 16.13), respectively. This implies that patients with initial VF had approximately 15-fold higher increased chance of favorable neurological outcomes than patients without initial VF. The likelihood that the initial recorded rhythm will be VF depends on the time interval since the onset of arrest [14]. When cardiac arrest is witnessed by EMS, as the time interval from collapse to the recording of initial cardiac rhythm may be minimum, the rates of initial VF rhythm will be increased. In the present study, the rate of initial VF rhythm in patients with EMS-witnessed arrest was higher than that in patients with non-EMS-witnessed arrest (7.6% (1408/18,601) versus 5.8% (22,088/379,519), *P* <0.0001). Accordingly, initial VF rhythm and EMS-witnessed arrest seemed to be closely associated with outcomes after OHCA without prehospital ROSC.

The second most important factor was age <65 years. Many studies have shown that the overall prognosis for survival is worse for older patients [15-18]. However, it is unknown whether comorbid disease is responsible for worse prognosis in elderly patients with OHCA [19]. We did not evaluate comorbid disease in this study because of lack of information in the database. Moreover, arrests in elderly persons less frequently present with VF as an initial rhythm [15]. This may also worsen the outcomes after OHCA. As termination of resuscitation in the field is not permitted in Japan, almost all patients with OHCA are transported to hospital. Accordingly, the mean age of patients transported to the hospital in the present study (mean 73.7 years, standard deviation 16.2) was higher than that in the United States [15] (mean 64.0 years, standard deviation 18.2) and European countries [16,18]. Therefore, the beneficial association of age with neurological outcomes may be reduced in other countries where termination of resuscitation in patients with refractory OHCA [5,6] is applied.

The third most important factor was EMS-witnessed arrest. In this special situation, patients have called for help before the cardiac arrest and thus, are able to receive good-quality resuscitation without delay. However, the percentage of EMS-witnessed arrest in our study subjects was only 4.7% (n = 18,601). An earlier observational study [20] indicated that over 60% of OHCA patients had symptoms prior to their cardiac arrest and 40% of such symptoms had been manifested at least several minutes before cardiac arrest. Therefore, the importance of early recognition of the prior signs of subsequent cardiac arrest and early activation of the EMS systems should be more emphasized to increase the frequency of EMS-witnessed arrest and achieve favorable neurological outcomes.

The fourth most important factor was call-to-hospital arrival time. Although call-to-hospital arrival time is considered a surrogate to the overall length of prehospital CPR, this value is affected by many factors, including call-to-response time, CPR time at the site by EMS personnel, distance from the site of arrest to the hospital, and traffic congestion. In Japan, living in the rural area was associated with an independent risk of delay in ambulance response, and a low survival-rate in cases of OHCA [8]. Although call-to-response time was an independent factor associated with 1-month CPC 1 to 2, the adjusted OR was less than that of call-to-hospital arrival time in our analytic model (Table 3). This implies that early transport to the hospital from the site is essential for OHCA patients without prehospital ROSC and may enable the implementation of more aggressive medications, such as assisted extracorporeal life support [21,22] followed by mild therapeutic hypothermia [23,24].

### Study limitations

The potential limitations of the current analysis are as follows. First, our database lacked detailed data to make further risk adjustment for outcomes, for example, comorbid disease of patients, location where the OHCA occurred, quality of EMS personnel, the degree of regional differences among EMS centers, in-hospital medication, and the availability of specialists in emergency care (cardiologists). These deficient data were associated with our study design of a retrospective record review. Second, the categories for age, call-to-response time, and call-to-hospital arrival time were made on the basis of the IQR of each variable. However, such division could not be generalized to other countries with different EMS systems. Third, although we used a uniform data collection procedure on the basis of the Utstein-style guidelines for reporting cardiac arrest, a large sample size, and a population-based design, we cannot exclude the possibility of uncontrolled confounders. Fourth, as with all epidemiological studies, the integrity, validity, and ascertainment bias of the data were potential limitations.

## Conclusions

In OHCA patients transported to hospitals without a prehospital ROSC, nine prehospital factors were significantly associated with 1-month favorable neurological outcomes. Of those, four are crucial key factors: (1) initial non-asystole rhythm (VF, pulseless VT, and PEA); (2) age <65 years; (3) EMS-witnessed arrest; and (4) call-to-hospital arrival time <24 minutes.

## Key messages

• We determined the prehospital factors that influence 1-month favorable neurological outcomes in patients transported to hospitals without a prehospital ROSC after an OHCA, using a prospectively recorded nationwide Utstein-style Japanese database.

• Nine prehospital factors were independently associated with increased odds of 1-month favorable neurological outcomes: (1) initial non-asystole rhythm (VF, pulseless VT, and PEA); (2) age <65 years; (3) arrest witnessed by EMS personnel; (4) call-to-hospital arrival time <24 minutes; (5) arrest witnessed by any layperson; (6) physician-staffed ambulance; (7) call-to-response time <5 minutes; (8) prehospital shock delivery; and (9) presumed cardiac cause.

• When four crucial key factors (with an adjusted OR of >2.0 in the multivariate logistic regression model for 1-month CPC 1 to 2: initial non-asystole rhythm, age <65 years, EMS-witnessed arrest, and call-to-hospital arrival time < 24 minutes) were present, the rates of 1-month CPC 1 to 2 and 1-month survival were 16.1% and 23.2% in initial VF, 8.3% and 16.7% in pulseless VT, and 3.8% and 9.4% in PEA, respectively.

## Abbreviations

CPC: Cerebral performance category; CPR: Cardiopulmonary resuscitation; ELST: Emergency life-saving technician; EMS: Emergency medical services; FDMA: Fire and Disaster Management Agency; OHCA: Out-of-hospital cardiac arrest; OR: Odds ratio; PEA: Pulseless electrical activity; ROSC: Return of spontaneous circulation; VF: Ventricular fibrillation; VT: Ventricular tachycardia.

## Competing interests

The authors declare that they have no competing interests.

## Authors’ contributions

YG and TM designed the study. YG, TM, and YN conducted data cleaning. YG and YN analyzed the data. YG drafted the manuscript, and YN, and TM contributed substantially to its revision. YG takes responsibility for the paper as a whole. All authors approved the manuscript before submission.
